# Basic resuscitation skills of medical students – a monocenter randomized simulation trial

**DOI:** 10.3205/zma001512

**Published:** 2021-11-15

**Authors:** Cara Bülow, Stella-Karolin Krispin, Franziska Lehmanski, Grit Spalding, Anja Haase-Fielitz, Christian Butter, Jonathan Nübel

**Affiliations:** 1 Medizinische Hochschule Brandenburg (MHB), Neuruppin, Germany; 2 Immanuel Klinikum Bernau, Herzzentrum Brandenburg, Zentrale Notaufnahme, Bernau bei Berlin, Germany; 3 Medizinische Hochschule Brandenburg (MHB), Hochschulklinikum, Neuruppin, Germany; 4 Immanuel Klinikum Bernau, Herzzentrum Brandenburg, Abteilung for Kardiologie, Bernau bei Berlin, Germany; 5 Otto-von-Guericke-Universität Magdeburg, Medizinische Fakultät, Institut für Sozialmedizin und Gesundheitssystemforschung, Magdeburg, Germany

**Keywords:** resuscitation, resuscitation competence, medical students, model course of study

## Abstract

**Objective:** The aim of this study was to evaluate resuscitation skills, defined as recognition of resuscitation situations and performance of Basic Life Support (BLS) in students at the Brandenburg Model Medical School (BMM).

**Methods: **Participating students (n=102) were randomized to different simulation scenarios: unconscious person with physiological breathing (15/min), gasping (<10/min) and apnea (resuscitation dummy AmbuMan^®^ Wireless with electronic recording). Primary endpoint was the proportion of students with correct decision for or against resuscitation. Secondary endpoint was resuscitation quality, self-assessment, and prior resuscitation experience. The latter two were assessed by questionnaire prior to the simulated situation.

**Results: **Overall, there was a high risk for incorrectly omitted or incorrectly performed resuscitation (OR 3.4 [95% CI 1.4-8.1] p=0.005. The highest probability of error occurred in the unconsciousness and gasping groups. 22.3% of all performed resuscitations where at the same time indicated and reached the European Resuscitation Council recommendations for compression frequency, pressure depth and where as well = 90% relieved. A particularly large discrepancy emerged between participants' self-assessment of being prepared for a resuscitation situation by medical school and their actual documented resuscitation competence.

**Conclusion: **The present data indicate significant uncertainty among students in recognizing a resuscitation situation. Even in curricula with a high proportion of practice and a high degree of students with completed vocational training in health care, resuscitation competence is poor.

## Zusammenfassung

**Zielsetzung: **Ziel der Untersuchung war die Analyse der Reanimationskompetenz definiert als Indikationserkennung und Durchführung des Basic Life Support (BLS) von Studierenden des Brandenburger Modellstudiengangs Medizin (BMM).

**Methodik: **Die teilnehmenden Studierenden (n=102) wurden zu unterschiedlichen Simulationsszenarien randomisiert: bewusstlose Person mit physiologischer Atmung (15/min), Schnappatmung (<10/min) und Apnoe (Reanimationsdummy AmbuMan^®^ Wireless mit elektronischer Aufzeichnung). Primärer Endpunkt war der Anteil an Studierenden mit korrekter Entscheidung für oder gegen eine Reanimation. Sekundärer Endpunkt war die Reanimationsgüte, Selbsteinschätzung und Reanimationsvorerfahrung. Letztere beiden wurden vor Studienbeginn durch einen Fragebogen erfasst.

**Ergebnisse:** Insgesamt bestand ein hohes Risiko für eine falsch unterlassene oder fälschlicherweise durchgeführte Reanimation (OR 3,4 [95% KI 1,4-8,1] p=0,005. Die größte Irrtumswahrscheinlichkeit ergab sich bei Bewußtlosigkeit und Schnappatmung. 22,3% aller indizierten und durchgeführten Reanimationen erreichten die vom European Resuscitation Council empfohlene Kompressionsfrequenz, Drucktiefe sowie 90% Kompressionsentlastung. Eine besonders große Diskrepanz ergab sich zwischen der Selbsteinschätzung der Teilnehmenden, durch die universitäre Lehre auf eine Reanimationssituation vorbereitet zu sein und ihrer tatsächlich dokumentierten Reanimationskompetenz.

**Schlussfolgerung: **Die vorliegenden Daten weisen auf deutliche Unsicherheit der Studierenden beim Erkennen einer Reanimationssituation hin. Selbst in Curricula mit hohem Praxisanteil und hohem Grad an Studierenden mit medizinischer Berufsausbildung ist die Reanimationskompetenz mangelhaft.

## Introduction

In cardiac arrest, preservation of victims’ physical and cognitive functions depends on early and correct assessment of the situation and high-quality chest compressions [1], [2], [3]. Often laypersons manage neither one nor the other [4], [5], [6]. As a competency-based objective for professional training in medical school, the Institute for Medical and Pharmaceutical Examination Questions (IMPP) addresses this skill in the “National Competence-Based Learning Objectives for Undergraduate Medical Education” (NKLM). Medical students should be able to *“explain and apply the basic life support algorithm (VII.4-03.2.1)”* [http://www.nklm.de].

Although medical students appear to have solid theoretical knowledge of correctly performed resuscitation by the time they graduate [7], they are often unable to confidently distinguish between sufficient and insufficient respiration [8]. The present study investigates whether medical students in BMM can reliably indicate and perform guideline-compliant chest compressions before and after the first part of their professional training.

## Methods

In a randomized trial, students within the participating semesters were randomized to different simulated scenarios (randomizer.org): “unconscious person with physiological breathing” (15/min), “gasping” (<10/min) or “apnea” (resuscitation dummy: AmbuMan^®^ Wireless with electronic recording). In addition, biographical data, previous experience, and self-assessment of all participants were recorded. The primary endpoint was the proportion of students with a correct decision for or against resuscitation within their simulated resuscitation scenario. Secondary endpoint was the quality of performed "compression only CPR". Resuscitation quality was assessed according to the European Resuscitation Council's recommended compression frequency, pressure depth, duration, and compression relief. 

For the analysis, a resuscitation was defined as *“high quality”* if the resuscitation was performed with correct indication and if recommended mean values in frequency and depth were achieved, with simultaneously more than 90% of the compressions at the correct pressure point and with sufficiently relieved. 

**Figure 1:**
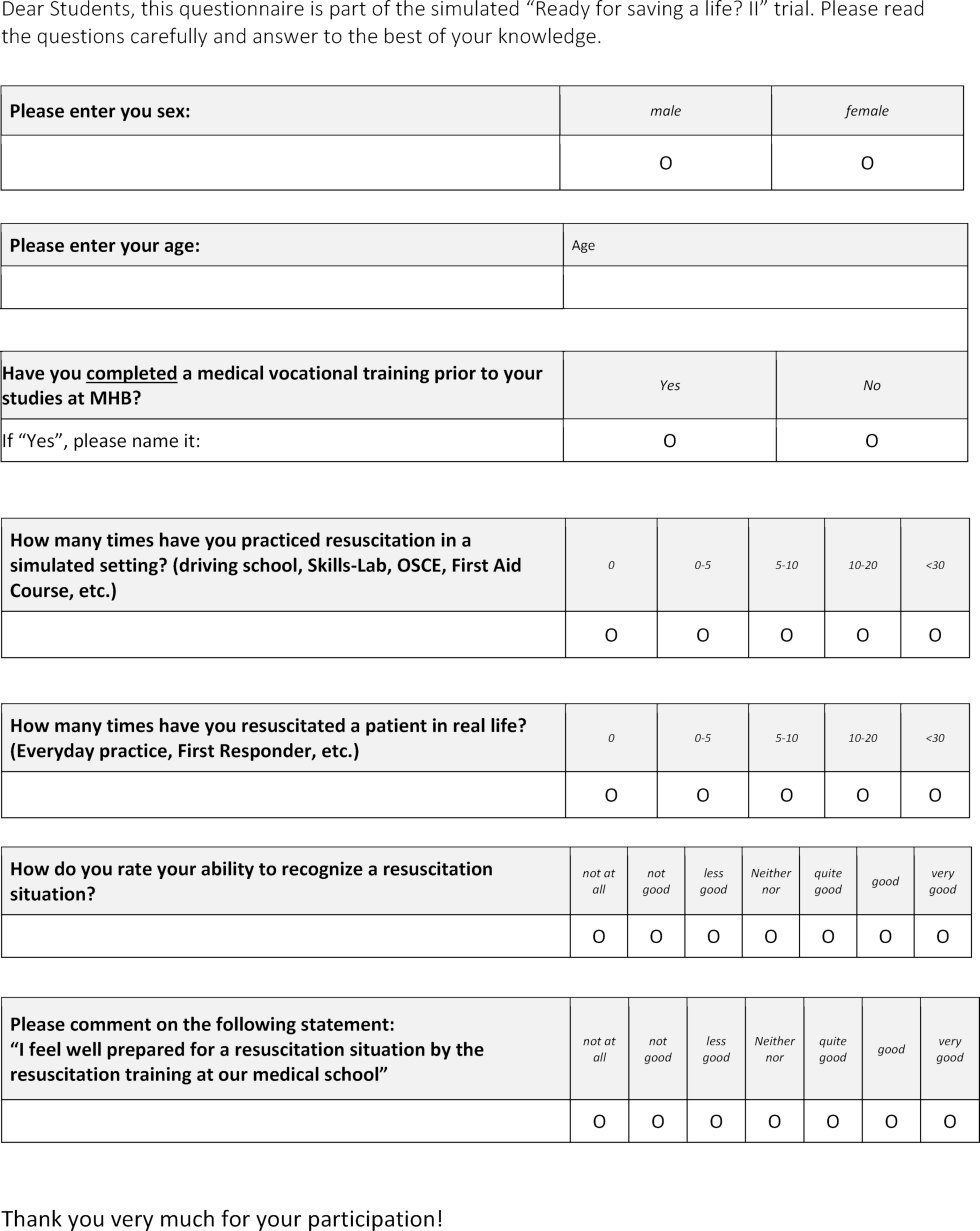
Questionnaire

Neither concrete instructions for action, nor group affiliation were available to the participants in advance. Participation in the extracurricular data collection was voluntary. Students in the 1^st^ semester had completed a one-week curricular emergency medicine training, 6^th^ semester students had completed another two-week training in addition [9]. The self-assessment questionnaire is available in figure 1 (Fig. 1). A positive ethics vote of the Brandenburg Medical School is available under file number E-01-20180724. For statistical analysis SPSS version 25 (IBM) was used. 

## Results

**Figure 2:**
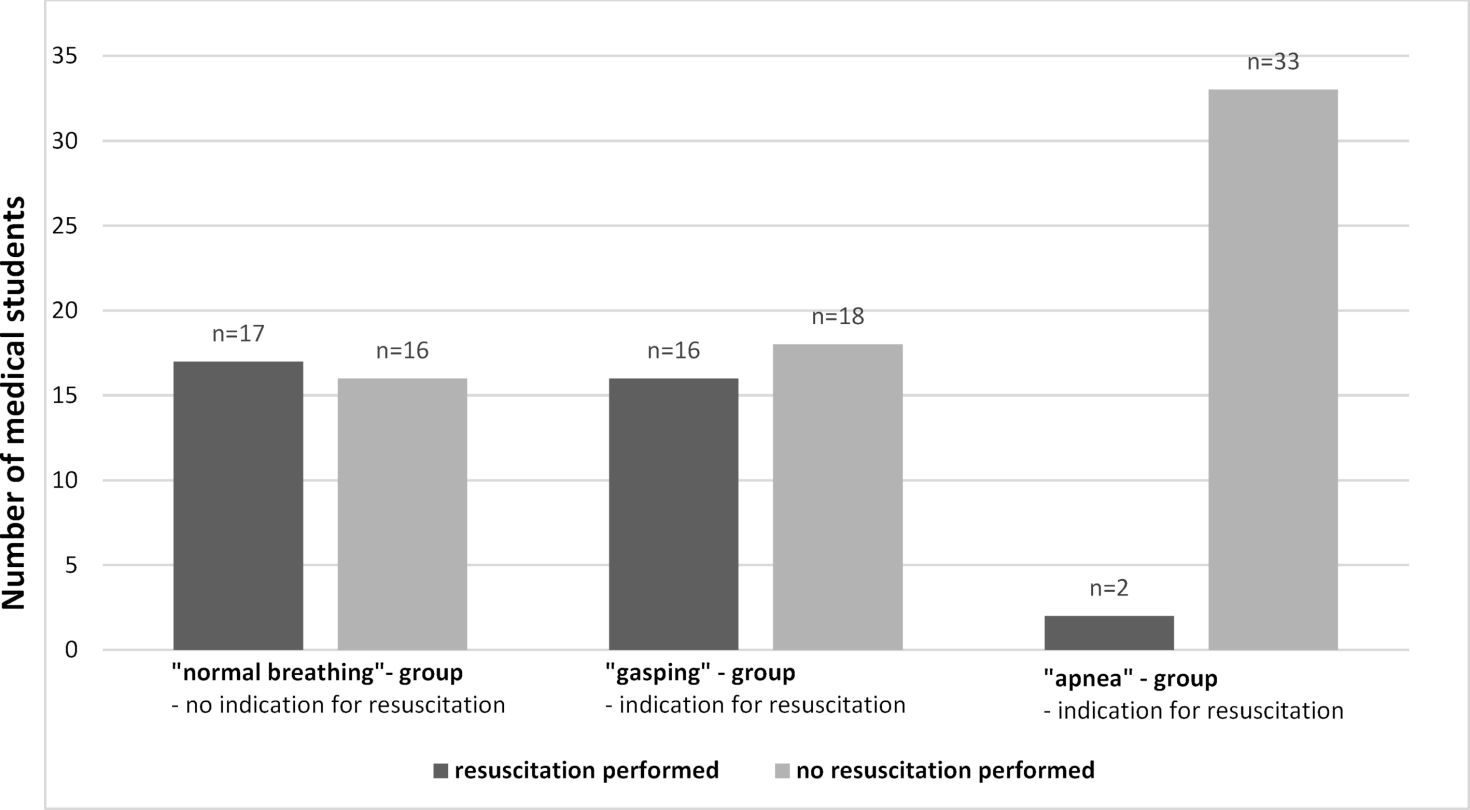
Number of medical students per group separated according to status of resuscitation. Of 102 participating students, n=33 were randomized to the physiologic breathing group, n=34 to the gasping group, and n=35 to the apnea group. The proportion of students who opted for resuscitation (black bar) or against resuscitation (gray bar) is shown separately.

The randomized study included n=102 medical students (n=63 women, n=31 1^st^ semester, n=71 6^th^ semester) (see table 1 (Tab. 1)). 69 students had a completed vocational training in health care prior to medical school. 59 students reported to have performed at least one resuscitation in real life. There were no statistically significant differences regarding resuscitation characteristics in-between the three groups (see table 2 (Tab. 2)). Overall, there was a high risk of incorrectly omitted or incorrectly performed resuscitation (OR 3.4 (95% CI 1.4-8.1) p=0.005 (see table 3 (Tab. 3)). The highest probability of error was found in the unconscious and gasping groups (see figure 2 (Fig. 2)). Even though no resuscitation was indicated, 48.5% initiated resuscitation in the simulated physiologic breathing group. Despite of an indication, resuscitation was initiated by only 52.9% of the students in case of gasping. In the apnea situation, 2 students omitted resuscitation. Overall, 15.3% (6^th^ semester n=11, 1^st^ semester, n=4) achieved high-quality resuscitation. Disregarding the correctness of the decision to resuscitate, 55% of the students achieved guideline-appropriate compression frequency, 60% achieved appropriate compression depth, and 71% achieved =90% compression relief. 31.6% of the participants who rated their competencies as “fairly good” or better overestimated their competencies to correctly recognize a resuscitation situation and did not act appropriately for their situation.

## Discussion

The present data indicates significant uncertainty among students in recognizing a resuscitation situation correctly. As reported in other studies, it was particularly difficult to differentiate breathing patterns [4], [8], [10], [11], [12]. Reliable recognition of “not-normal breathing” is particularly important, as gasping is present in about 40% of all cardiovascular arrests [13]. 

In case of doubt, its preferable starting BLS rather than doing none. Nevertheless, the indication should be based on comprehensible criteria and on the recommendations of the professional societies. 

Although the self-assessment of being prepared for a resuscitation situation seems to be better than in European-wide comparison [8], there is a particularly large discrepancy between the self-assessment of participants and their documented resuscitation competence in this study. This cognitive bias could be based on overestimation of abilities by those with low abilities (Kruger-Dunning effect) [14]. Reinforcement by supposed but insufficient experience (vocational-, CPR training) is conceivable and could amply [15]. 

Using technical feedback systems on compression quality is superior to standard training [16] and could also lead to better reflection of own competencies. Positive effects of targeted training on correctly indicate a resuscitation situation, have been reported [11]. Based on the present data this should be more addressed in resuscitation training. Medical students from other faculties, with a presumably smaller proportion of completed vocational training, may at best have similar practical resuscitation competence than shown here. Whether students should demonstrate high resuscitation competency to pass their professional training or course design and examinations needs to be modified, should be examined in multicenter, cross-faculty studies.

It should be noted that we chose the 90% threshold for the compression’s relief because instead, according to the ERC guideline, even a single improperly relieved compression would have met the definition of a not-high-quality resuscitation. Finally, the limitation of any simulation study must consider, which only represents reality to a certain extent, but emphasizes the importance of a practical analysis of resuscitation skills [17].

## Authorship

Shared authorship: Jonathan Nübel and PD Dr. Anja Haase-Fielitz

## Competing interests

The authors declare that they have no competing interests. 

## Figures and Tables

**Table 1:**
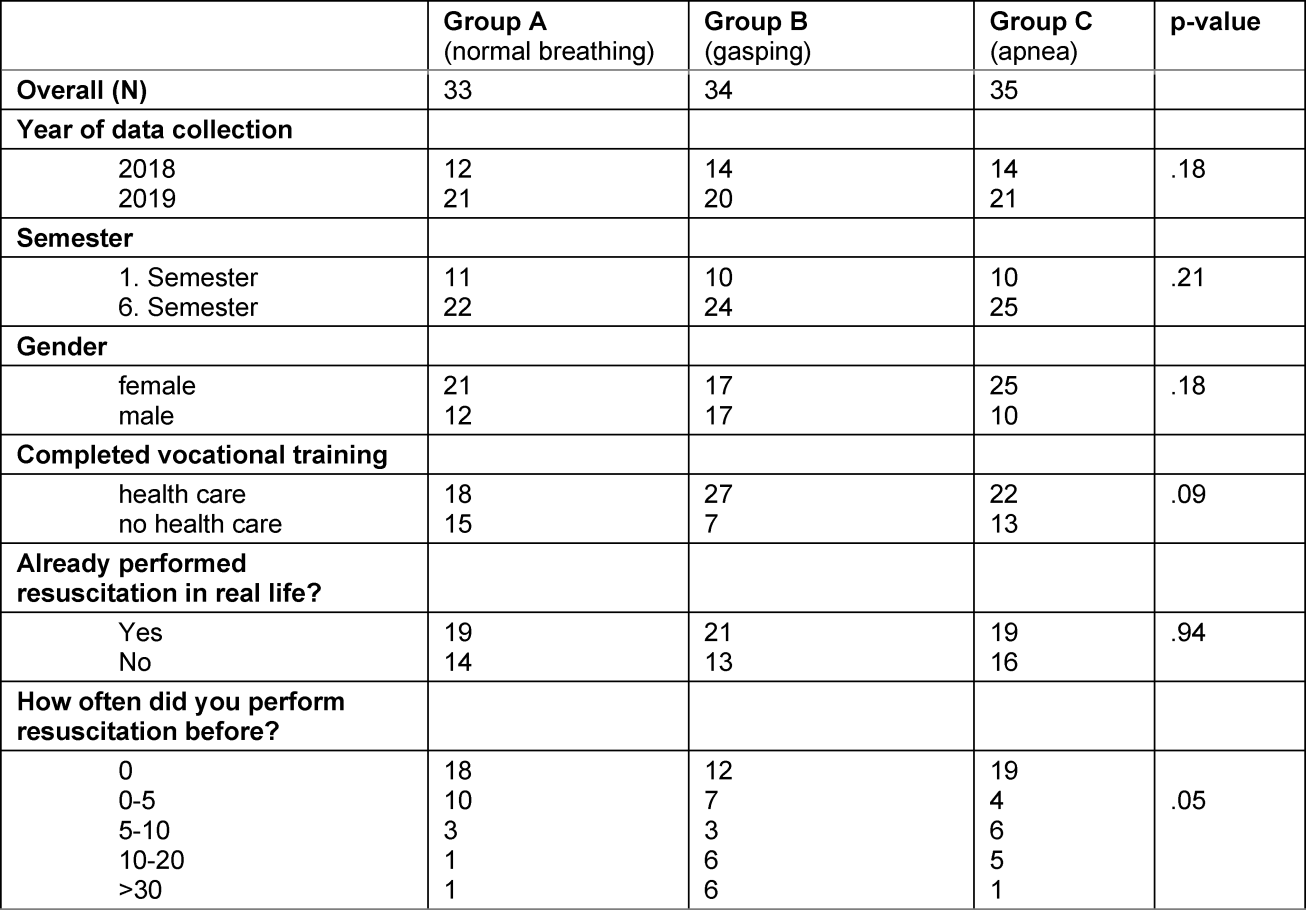
Demographics

**Table 2:**
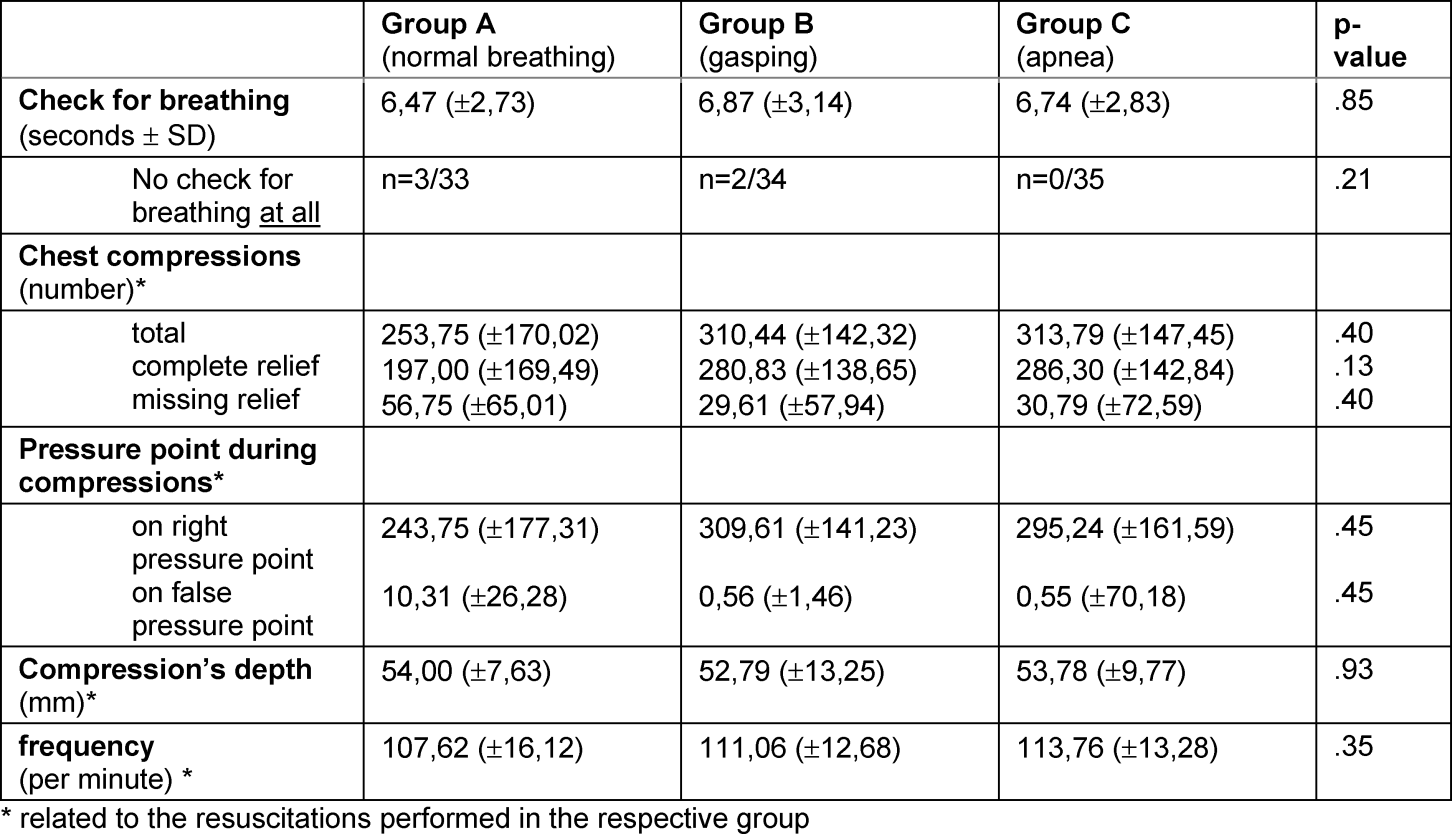
CPR characteristics

**Table 3:**
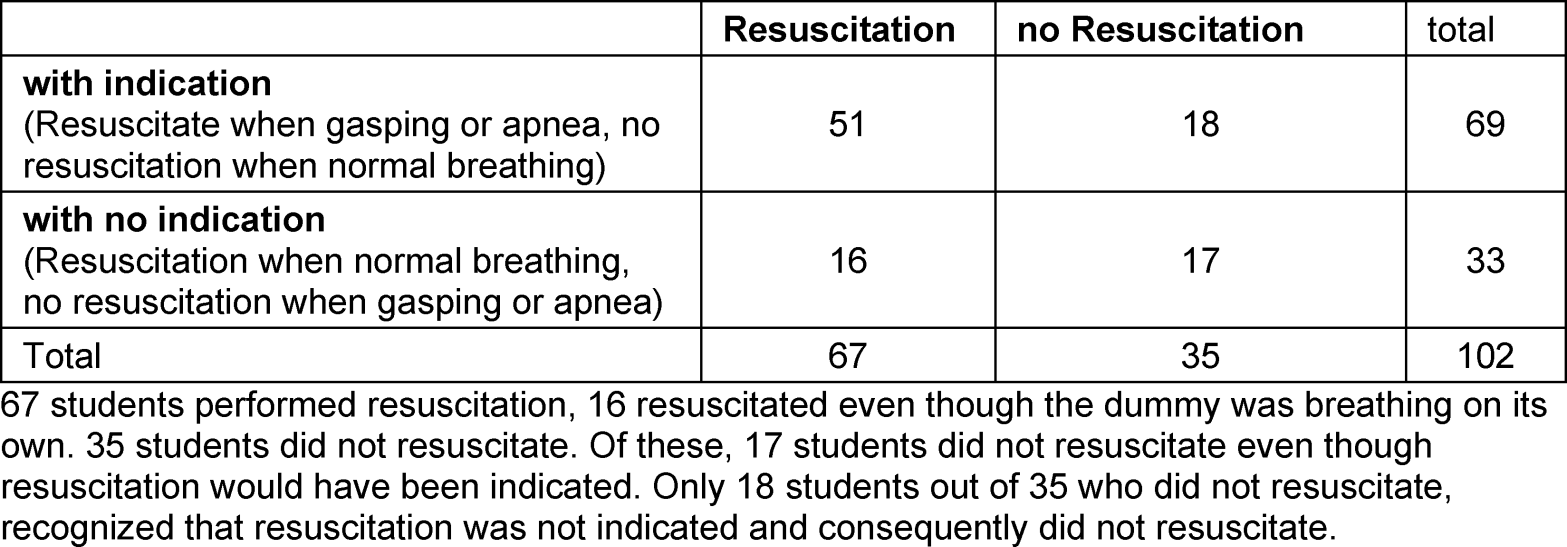
Indication for resuscitation
